# Long-term results of a clinical trial comparing isolated vaginal stimulation with combined treatment for women with stress incontinence

**DOI:** 10.1590/S1679-45082014AO2866

**Published:** 2014

**Authors:** Maria Cláudia Bicudo Fürst, Rafaela Rosalba de Mendonça, Alexandre Oliveira Rodrigues, Leandro Luongo de Matos, Antônio Carlos Lima Pompeo, Carlos Alberto Bezerra

**Affiliations:** 1 Faculdade de Medicina do ABC, Santo André, SP, Brazil.

**Keywords:** Urinary incontinence/rehabilitation, Electric stimulation, Urinary incontinence, stress

## Abstract

**Objective:**

To determine the efficacy of stress urinary incontinence treatments adding pelvic floor muscle training to vaginal electrical stimulation.

**Methods:**

Forty-eight women with stress urinary incontinence were randomized into 2 groups: 24 underwent isolated vaginal electrical stimulation, and 24 vaginal electrical stimulation plus pelvic floor muscle training. History, physical examination, voiding diary, perineum strength test, and urodynamic study were assessed. Comparisons were made for adherence to treatment, muscle strength improvement, urinary symptoms, and degree of satisfaction immediately, 12 and 96 months after treatment.

**Results:**

Patients' degree of satisfaction on vaginal electrical stimulation, and on vaginal electrical stimulation plus pelvic floor muscle training immediately, 12 and 96 months post treatment, were, respectively: 88.2% *versus* 88.9% 64.7% *versus* 61.1% and 42.9% *versus* 28.6% (p>0.05).

**Conclusion:**

Vaginal electrical stimulation associated to pelvic floor muscle training did not show better results than vaginal electrical stimulation alone.

## RESUMO

**Objetivo:**

Determinar a eficácia da eletroestimulação vaginal combinada com treinamento muscular do assoalho pélvico para o tratamento da incontinência urinária de esforço.

**Métodos:**

Um total de 48 mulheres com incontinência urinária de esforço foi randomizado em 2 grupos, sendo 24 submetidas a eletroestimulação vaginal isolada e 24 a eletroestimulação vaginal e treinamento muscular do assoalho pélvico. Foram avaliados anamnese, exame físico, diário miccional, força perineal e urodinâmica. Compararam-se a adesão ao tratamento, a melhora da força muscular perineal e dos sintomas urinários, e o grau de satisfação imediatamente, 12 e 96 meses após o tratamento.

**Resultados:**

O grau de satisfação das pacientes no grupo da eletroestimulação isolada e do segundo grupo, imediatamente, com 12 e com 96 meses foi, respectivamente, 88,2% *versus* 88,9% 64,7% *versus* 61,1% e 42,9% *versus* 28,6% (p>0,05).

**Conclusão:**

A eletroestimulação vaginal associada ao treinamento muscular do assoalho pélvico não foi mais eficaz do que a eletroestimulação isolada.

## INTRODUCTION

Urinary incontinence (UI) is a common problem that affects women at any age. Although the UI prevalence in women varies from 4.5 to 53%, less than 50% of patients seek medical care.^(1)^ The estimated annual cost for UI treatment is approximately U$11 billion in the United States.^(2)^

The most common type of UI in women is stress urinary incontinence (SUI), defined as the involuntary loss of urine due to conditions that determine increased intra-abdominal pressure. Risk factors include vaginal delivery, age, obesity and increased physical effort.^(2)^

SUI treatment can be surgical or conservative.^(3)^ The goal of conservative treatment is to strengthen the pelvic floor muscles through several techniques, including biofeedback, electrical stimulation (ES) and pelvic floor muscle training (PFMT) alone^(4)^ or in association.^(5,6)^ Several clinical questions addressing physiotherapy techniques need answer in current literature. Protocols need standardization and there is lack of randomized trials and longer follow-up reports.

## OBJECTIVE

The aim of this study was to determine the efficacy of adding pelvic floor muscle training to vaginal electrical stimulation in the treatment of stress urinary incontinence in women and to report long-term (8 years) follow-up.

## METHODS

It was a prospective, randomized study carried out between August 2000 and September 2002, with 48 women with clinical and urodynamic SUI recruited from the Faculdade de Medicina do ABC, Santo André (SP, Brazil) and randomly assigned to each arm of the study protocol. Randomization was based on a random number table and allocation was done by a third part. This was performed after participants signed an Informed Consent Form, which had been previously accepted by our institutional Ethics Committee (protocol 057/2002).

Literature review demonstrated satisfaction rates for VES ranging from 48% to 94%, similar to those seen on clinical practice. For sample estimation, we selected the value of 90%.^(7–9)^ The combined treatment with PFMT was estimated as 65% or greater.^(5)^ Assuming a test of 80% power to detect a difference between both groups and 5% of significance level, with use of a two-sided test, we calculated a sample of 16 patients in each group. Estimating eventual losses, a total of 24 patients cases were included.^(10)^

Patients were divided into 2 groups: 24 patients underwent VES alone and 24 VES plus PFMT. Initially, a clinical assessment was used to obtain personal data, obstetric, gynecological and family history, urinary symptoms and subjective analysis of leak of urine. Patients underwent a physical examination to assess pelvic organ prolapse and body mass index (BMI). Patients with a history of surgical treatment for SUI, pelvic reconstruction and hysterectomy were included. Comparative demographic clinical data of patients who completed treatment protocol are shown in table 1.

**Table 1:** Group characteristics

				Group 1 n=17	Group 2 n=18	p-value
Personal data						
	Age+			49 (±11.0)	50.2 (±10.7)	0.753**
	Body mass index+			28.6 (±3.54)	25.3 (±4.64)	0,049**
	Parity+			4 (±2.26)	4 (±1.95)	0.938**
	Vaginal delivery+			2.3 (±2.22)	3 (±2.23)	0.321**
	Forceps delivery+			0.18 (±0.39)	0	0.386*
	Caesarean delivery+			1.0 (±1.84)	0.61 (±1.03)	0.443**
	Pelvic surgery++			2 (11.8)	7 (38.9)	0.121¤¤
	Hysterectomy++			4 (23.5)	5 (27.8)	1.000¤¤
	Incontinence surgery++			2 (11.8)	7 (38.9)	0.121¤¤
	Menopause++			7 (41.2)	13 (72.2)	0.092¤¤
	Hormonal reposition++			3 (17.6)	7 (38.2)	0.264¤¤
Urinary symptoms						
	Pad need++			9 (47.2)	10 (52.6)	0.730¤
	Urgency++			11 (68.8)	10 (58.8)	0.721¤¤
	Sterss incontinence++			17 (100)	18 (100)	1.000¤
	Urge incontinence++			11 (64.7)	11 (61.1)	1.000¤
	Nocturia++			10 (58.8)	11 (61.1)	0.890¤
	Disuria++			2 (11.8)	3 (17.6)	1.000¤¤
Physical examination						
	Perineum integrity++			5 (33.3)	12 (66.7)	0.084¤¤
		Cystocele++		13 (76.4)	13 (72.2)	0.717¤
			GI	5 (38.5)	7 (53.8)	
			GII	7 (53.8)	5 (38.5)	
			GIII	1 (7.7)	1 (7.7)	
		Rectocele++		9 (52.9)	10 (55.5)	0.556¤
			GI	5 (55.6)	4 (40)	
			GII	4 (44.4)	5 (50)	
			GIII	0	1 (10)	

+ Average ± standard deviation;

++ number of cases and percentage;

* Mann-Whitney U-test;

** Student's *t* test;

¤ q test;

¤¤ Fisher's exact test.

Group 1: vaginal electrical stimulation; Group 2: vaginal electrical stimulation + pelvic floor muscle training.

The patients were instructed to complete a voiding diary by registering for 1 week their daily urinary frequency, number of episodes of UI and nocturnal micturition. The diary was obtained in the week before beginning therapy and then after 3 months at the end of the intervention.

Objective evaluation of perineum strength was carried out using intravaginal cones (FemTone^®^ – Coloplast). Patients were instructed to introduce and maintain cones with weights progressively higher inside their vagina. Five weights (20g; 32.5g; 45g; 57.5g and 70g) were used. The greatest weight that a patient could maintain for 2 minutes was noted (evaluation at rest). Then patients repeated the procedure, moving through the examination room with different cones, to define the one they could sustain (evaluation in motion). This evaluation was repeated after treatment and changes were noted. The urodynamic examination was performed at the beginning of the study (Table 2).

**Table 2:** Urodynamic findings

	Group 1 n=17	Group 2 n=18	Valor de p
Uroflowmetry+	17.50 (±1.291)	14.6 (±5.05)	0.308*
Total voided volume+	201.67 (±105.159))	143.0 (±21.21)	0,512*
Postvoid residual+	0	112.5 (±225.0)	0.437*
Involuntary detrusor Contraction++	2 (11.8%)	4 (22.1%)	0.645**
Abdominal leak point Pressure+	80.86 (±47,57)	94.38 (±45.00)	0.456*
Flow+	13.87 (±8.05)	20.0 (±9.89)	0.497*
Final residual+	0	0	-

+ Average ± standard deviation;

++ number of cases and percentage;

* Student's *t* test;

** Fisher's exact test.

Group 1: vaginal electrical stimulation; Group 2: vaginal electrical stimulation + pelvic floor muscle training.

The treatment protocol proposed for 3 months consisted of VES and PFMT. VES was performed with vaginal probe and stimulation device (Dualpex 961^®^ - Quark Co.) at the outpatient unit care, under physical therapist supervision. All patients underwent 2 weekly sessions of 30 minutes stimulation with frequencies of 4Hz (15 minutes, 1ms pulse) and 50Hz (15 minutes, 700*µ*s pulse), fixed intensity (20mA) and 4 seconds stimulation *versus* 8 seconds rest.

PFMT consisted of exercises carried out in an individualized program designed by the physical therapist and with repeated contraction/relaxation of pelvic floor muscles, during 30 minutes in the unit care. Training was performed in the day alternate to VES twice a week. Patients received instructions about pelvic floor anatomy by the same doctor (CAB) and were assisted during exercises by a physical therapist. They did not receive instructions to do home exercises for the pelvic floor, but suggested to work accessory muscles (adductors, extensors, abductors and abdominal muscle).

Outcome measures included adherence to conservative treatment, the effect on perineum muscle strength at rest and in movement, improvement in urinary symptoms or episodes of incontinence and also satisfaction after treatment completion. The degree of satisfaction was based on patient's perception of the need or not to repeat or change treatment.

At the end of the treatment all women were encouraged to maintain follow-up and report the exact time the satisfaction persisted. No other therapy was planned.

After 96 months, in order to analyze the long-term results of that population studied but not followed by the protocol, the charts were reviewed regarding reported data. Then, patients were interviewed by phone or telegram to access urinary symptoms, persistence of incontinence, pad use and satisfaction. Only the patients who had not received any additional therapy were considered for analysis.

The statistical analysis package *Statistical Package for the Social Science* (SPSS), version 13.0 (Illinois, USA), was used and the significance level considered was 5% (p<0.05). Variable distribution as parametric or not parametric was determined by Kolmogorov-Smirnov test. Variables were represented by absolute frequency (n), relative frequency (%) and arithmetic mean and standard-deviation. Comparison between classified variables was assessed by the Fisher exact test and χ^2^ test. Comparison between averages of continuous parametric variables was performed through Student's *t* test, paired *t* test and Analysis of Variance (ANOVA) and by Mann-Whitney *U* test for not parametric variables. For correlation studies, Pearson correlation coefficient test was applied.

## RESULTS

**Figure 1:**
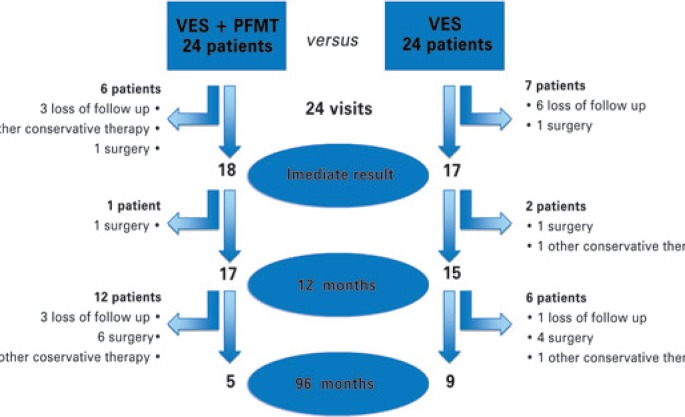
Follow-up assignment VES: vaginal electrical stimulation; PFMT: pelvic floor muscle training.

At the beginning, 48 patients were recruited and randomized into 2 groups, but only 35 completed the entire program over the 3 months, in 24 visits. The VES Group continued with 17 patients: 6 quit the program because of dissatisfaction with the results and 1 was submitted to surgery for incontinence, once it was proposed by other public health service. The other group continued with 18 patients, but 3 quitted for dissatisfaction, 1 had surgery and 2 decided for another conservative treatment proposed by other health services (Figure 1).

The average age of patients included was 49.6±10.60 years, with no difference between groups. The groups were homogeneous for analysis. There was no significant influence of age, parity, vaginal and forceps delivery or caesarean section, hormone status, urinary symptoms, presence of pelvic organ prolapse or previous surgery (Table 1). Only BMI showed significant difference, although not clinical significant because both groups showed overweight.

**Figure 2:**
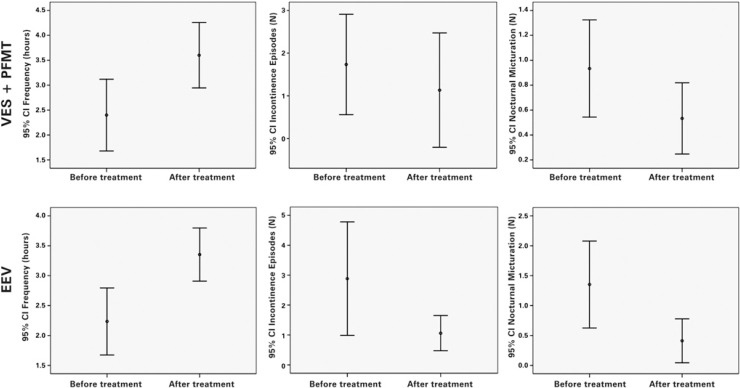
Voiding dairy VES: vaginal electrical stimulation; PFMT: pelvic floor muscle training; 95%CI: 95% confidence interval.

The perineum strength evaluated at rest and in motion for both groups showed no difference in the initial and final evaluation, which means there was neither increase nor reduction of the initial strength. Considering the symptoms reported on the voiding diary before treatment and after 3 months there was significant increase on time between micturition in VES+PMFT and VES Groups, 2.40±1.29 to 3.60±1.83 hours (p<0.0001; paired *t* test) and 2,24±1.09 to 3.35±0.86 hours (p<0.0001; paired *t* test), respectively. A significant reduction of nocturnal micturition was observed in both groups 0.93±0.704 to 0.53±0.516 episodes (p=0.028; paired *t* test) for VES+PFMT and 1.35±1.41 to 0.41±0.71 episodes (p=0.009; paired *t* test). For leak episodes, a significant reduction for both was also observed, on VES+PFMT Group 1.73±2.12 to 1.13±2.41 episodes (p=0.014; paired *t* test) and on VES 2.88±3.69 to 1.06±1.14 episodes (p=0.038; paired *t* test) (Figure 2). The final voiding diary findings were compared among groups and no statistical difference was observed into all parameters (p>0.05; *t* test). Therefore both have the same final effect on urinary symptoms by reducing the frequency and improving incontinence and nocturia.

**Figure 3:**
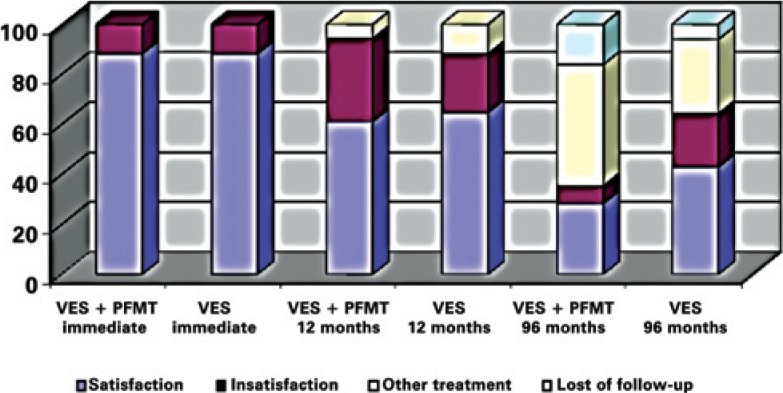
Satisfaction levels over all follow-up periods VES: vaginal electrical stimulation; PFMT: pelvic floor muscle training.

Satisfaction analyzed in both groups and among all intervals showed no statistical difference on immediate (p=1.000), 12 months (p=0.712) and 96 months (p=1.000; Fisher's exact test) (Figure 3).

When all initial characteristics related to the degree of satisfaction were compared, no statistical difference was found among perineum integrity, cystocele, rectocele, previous surgery, menopause or hormonal status, parity or type of delivery, urinary symptoms on voiding diary, urodynamic findings and perineum strength.

Patients remained satisfied for mean time of 43.35±10.46 and 27.67±8.13 months on VES and VES+PFMT Groups, respectively, with no statistical difference (p=0.660; Mann Whitney test). When considered all the initial variables, over time of sustained satisfaction no difference in both groups was found on parity, physical characteristics, previous surgery, hormonal status, perineal strength and urodynamic findings. However, there was a positive correlation (r=+0.56) on caesarean section with statistical difference (p=0.019; Pearson's correlation test) and a negative correlation (r=-0.58) on vaginal delivery with also statistical difference in Group 1 (p=0.013; Pearson's correlation test); such difference not observed on the other group.

Finally, comparing the initial urinary symptoms with those reported on the interview over 96 months we found no statistical difference among groups with regard to pad need, urgency, nocturia and stress incontinence. However, when comparing patients who had chosen another treatment, mainly surgery, over the follow-up period, we found that patients who initially presented with urge incontinence (p=0.046; Fisher's exact test) or more vaginal delivery (p=0.002; Student's *t* test: 1.29±1.44 deliveries against 3.43±1.91) were significantly more prompt to choose a new treatment. There was no statistical difference among VES *versus* VES+PFMT groups (p=0.257; Fisher's exact test).

## DISCUSSION

Urinary incontinence conservative treatment provides a minimally invasive approach; however its success depends on both motivation and commitment of the patient and also on the involvement of a multidisciplinary team.^(11)^

Kegel was the first to describe an exercise program for training pelvic floor muscles in the treatment of UI. The cure rate was 84% of patients.^(12)^

Electrical stimulation of the pelvic floor was first described in 1963^(13)^ and presented conflicting results, due to considerable variation in the type of stimulator, electrical parameters and methods used to analyze the results.^(14)^ Cure rates ranged from 30 to 50%, and there was improvement of urinary symptoms in 6 to 90% of patients.^(7–9)^

The precisely mechanism of these treatments is not known. Perineum exercises seem to produce recruitment of fast twitch muscle fibers, improving the reflex of perineum contraction, and increase the tone of slow twitch fibers, improving urethral and bladder support at rest. ES has unclear mechanisms. The stimulation electrodes cause ES of the pudendal nerve, which activates the pelvic floor muscles and inhibits the detrusor contraction.^(15)^ Low frequencies (5 to 10Hz) inhibit detrusor contractions and higher frequencies (45 to 50Hz) stimulate contraction of skeletal muscle.^(16)^

PFMT can be considered the first-line management of conservative programs for women with SUI. ES and vaginal cones should be offered when patients are unable to contract their pelvic floor muscle.^(17)^ Considering that more than 30% of incontinent women cannot contract their pelvic floor muscle correctly,^(18)^ such therapies seem to be an attractive option.

The association of methods is justified by the learning phase of perineum exercises, when patients tend to increase urinary leak and increase the rates of noncompliance.^(5)^ The association of biofeedback may add benefit to PFMT.^(19,20)^ Amaro et al.^(5)^ using vaginal stimulation and perineum exercises observed improvement in 67% of patients. ES helps patients to better recognize the target muscle of treatment, thus facilitating the use of the exercises.^(5)^

Exercise of the pelvic floor muscles was compared with ES in randomized controlled trials.^(21–25)^ In two studies, exercise alone was significantly more effective than ES.^(21,22)^ In others, there were no statistically significant difference.^(24–27)^

Several pitfalls are common in most trials of conservative treatment in current literature. Usually, the studied population is small, treatment protocols are extremely variable and not standardized, and randomized trials with long-term results are lacking. The current study addressed some of these problems and brings into discussion some new information.

We observed that the addition of perineum exercises does not improve the effectiveness of ES as an isolated method. Both treatments acted significantly to reduce frequency and improve incontinence and nocturia on the final voiding diary three months after treatment.

Subjective analysis on the degree of satisfaction observed immediately, after 12 months and 96 months, for VES and VES+PFMT, was 88.2 *versus* 88.9%; 64.7 *versus* 61.1%, and 42.9 *versus* 28.3%, respectively. None of the initial characteristics were considered an independent factor for the degree of satisfaction. In spite of worsening of the initial results, there was no difference between treatment groups during the entire follow-up, suggesting that using two methods neither improves the results immediately nor in the long term.

The literature data is very variable, with satisfaction rates ranging from 48 to 94%.^(7,9)^ The present study had good results in the short-term follow-up, however, an important failure rate was observed in the long-term subjective analysis.

In a systematic review,^(28)^ a total of 1,141 women were followed between 1 and 15 years. Losses to follow-up during the long-term period ranged between zero and 39%. Long-term adherence to PFMT varied between 10 and 70%. Five studies reported that the initial success rate on SUI was maintained. Long-term success based on responders to the original trial varied between 41 and 85%. Surgery rates varied between 4.9 and 58%.

Decrease in satisfaction among patients that initially presented urge incontinence or more vaginal delivery lead to more probability to choose another treatment, mainly surgery (p=0.046 and p=0.002). There was no statistical difference among groups (p=0.257).

It is not clear the exact reason for that, which could be only by chance. On the other hand, this could be due to the fact that patients did not improve their perineum strength whatever treatment was applied on the protocol. We cannot say whether this occurred as result of intrinsic characteristic of the patients or of the protocol method, but it made us to believe that patients with UI and/or multiple vaginal deliveries should undergo retreatment or new kind of therapies to achieve satisfaction (or at least should be advised of that risk).

Patients remained satisfied for 43.35 months on VES alone and 27.67 months on VES+PMFT with a significant positive correlation with caesarean section and significant negative correlation with vaginal delivery for the VES group.

Possible reasons for these discrepancies in results may be lack of standardization when measuring outcomes, small sample size, cultural and social aspects of population and differences in protocols. Data shows women remaining dissatisfied even with improvement of UI and individual responses varying markedly.^(29)^

We did not follow some recommendations such as using pad tests and quality of life questionnaires for local restrictions and transitory difficulties. These faults could have direct impact in our results. In addition, we lost a significant amount of participants during follow-up. However, our results are not quite different from other trials in terms of success rate.

It is difficult to explain the fact that patients who underwent the two treatments had no better results. One possibility is that ES alone, as would be expected with exercise alone, already offers the maximum degree of improvement patients could get and, so far, the association would not add any benefits.

Another possible explanation is that patients who underwent pelvic training did not perform the exercises properly and therefore had no additional benefit. This may occur due to low adherence to the exercise program, lack of understanding of anatomy and/or of instructions, and small sample size. This hypothesis is minimized with the active participation of physical therapists and with the compliance and motivation of our patients, but in fact, this question is yet unclear.

Finally, it could be argued that more precise information for each method could provide better results, especially considering objective analysis, like pad test or repeated urodynamic evaluation.

In our study the number of patients studied was too small to obtain more sound data and establish difference among groups. This may be subject of new future research opportunities. Although few patients completed the 96-month follow-up, the satisfaction sustained in some of them means that new investments on better protocols may avoid surgery in some cases.

## CONCLUSION

Pelvic floor rehabilitation by vaginal electrical stimulation, with or without exercise, had efficacy similar to literature data in the treatment of stress urinary incontinence. The addition of pelvic exercise did not improve the results compared to vaginal electrical stimulation alone even in long-term follow-up.
